# Identification of a new cell line permissive to porcine reproductive and respiratory syndrome virus infection and replication which is phenotypically distinct from MARC-145 cell line

**DOI:** 10.1186/1743-422X-9-267

**Published:** 2012-11-13

**Authors:** Chantale Provost, Jian Jun Jia, Nedzad Music, Cynthia Lévesque, Marie-Ève Lebel, Jérôme RE del Castillo, Mario Jacques, Carl A Gagnon

**Affiliations:** 1 Groupe de recherche sur les maladies infectieuses du porc (GREMIP), Centre de recherche en infectiologie porcine (CRIP), Faculté de médecine vétérinaire Université de Montréal, 3200 rue Sicotte, Saint-Hyacinthe, J2S 7C6, Québec, Canada; 2 Groupe de Recherche en Pharmacologie Animale du Québec (GREPAQ), Faculté de médecine vétérinaire Université de Montréal, 3200 rue Sicotte, Saint-Hyacinthe, Québec, J2S 7C6, Canada

**Keywords:** Porcine reproductive and respiratory syndrome virus, PRRSV, SJPL cells, Virus replication, Cell permissivity, Type 1 IFN, IFNγ, TNF-α, Cytokines

## Abstract

**Background:**

Airborne transmitted pathogens, such as porcine reproductive and respiratory syndrome virus (PRRSV), need to interact with host cells of the respiratory tract in order to be able to enter and disseminate in the host organism. Pulmonary alveolar macrophages (PAM) and MA104 derived monkey kidney MARC-145 cells are known to be permissive to PRRSV infection and replication and are the most studied cells in the literature. More recently, new cell lines developed to study PRRSV have been genetically modified to make them permissive to the virus. The SJPL cell line origin was initially reported to be epithelial cells of the respiratory tract of swine. Thus, the goal of this study was to determine if SJPL cells could support PRRSV infection and replication *in vitro*.

**Results:**

The SJPL cell growth was significantly slower than MARC-145 cell growth. The SJPL cells were found to express the CD151 protein but not the CD163 and neither the sialoadhesin PRRSV receptors. During the course of the present study, the SJPL cells have been reported to be of monkey origin. Nevertheless, SJPL cells were found to be permissive to PRRSV infection and replication even if the development of the cytopathic effect was delayed compared to PRRSV-infected MARC-145 cells. Following PRRSV replication, the amount of infectious viral particles produced in SJPL and MARC-145 infected cells was similar. The SJPL cells allowed the replication of several PRRSV North American strains and were almost efficient as MARC-145 cells for virus isolation. Interestingly, PRRSV is 8 to 16 times more sensitive to IFNα antiviral effect in SJPL cell in comparison to that in MARC-145 cells. PRRSV induced an increase in IFNβ mRNA and no up regulation of IFNα mRNA in both infected cell types. In addition, PRRSV induced an up regulation of IFNγ and TNF-α mRNAs only in infected MARC-145 cells.

**Conclusions:**

In conclusion, the SJPL cells are permissive to PRRSV. In addition, they are phenotypically different from MARC-145 cells and are an additional tool that could be used to study PRRSV pathogenesis mechanisms *in vitro*.

## Background

Porcine reproductive and respiratory syndrome (PRRS) is present worldwide and is economically speaking, one of the most important infectious diseases in swine production [1]. PRRS disease was first described in the United States in 1987 [2,3] and a few years later in the Netherlands [4]. The disease has many clinical manifestations but the two most prevalent are severe reproductive failure in sows and gilts (characterized by late-term abortions, an increased number of stillborns, mummified and weak-born pigs) [2,5] and respiratory problems in pigs of all ages associated with a non-specific lymphomononuclear interstitial pneumonitis [2,5,6].

The etiological agent, porcine reproductive and respiratory syndrome virus (PRRSV) was identified in 1991 by investigators in the Netherlands and shortly after in the USA [4,7,8]. PRRSV is an enveloped, single-stranded positive sense RNA virus, approximately 50–65 nm in diameter classified in the order *Nidovirales*, family *Arteriviridae*, genus *Arterivirus* along with equine arteritis virus (EAV), lactate dehydrogenase-elevating virus of mice (LDV), and simian hemorrhagic fever virus (SHFV) [7,9]. PRRSV genome is approximately 15 kb in length. The viral RNA genome is capped at the 5’ end and polyadenylated at the 3’ end and encodes at least ten open reading frames (ORFs) [10-12], each of which is expressed via the generation of a 3’-coterminal nested set of subgenomic (sg) mRNAs [13]. The virus is genetically, antigenically, and pathogenically heterogeneous [14,15]. PRRSV isolates are currently divided into two distinct genotypes, the European genotype (EU) or type I represented by the Lelystad virus (LV) and the North American genotype (NA) or type II represented by the ATCC VR-2332 strain [16].

PRRSV is known to have a very restricted cell tropism both *in vivo* and *in vitro*. *In vivo*, the virus infects mainly well-differentiated cells of the monocyte-macrophage lineage, in particular porcine alveolar macrophages (PAMs), the primary target cells of virus and interstitial macrophages in other tissues such as heart, thymus, spleen and Peyer's patches, hepatic sinusoids, renal medullary interstitium, and adrenal gland [17-20]. In addition to macrophages, PRRSV RNA and nucleocapsid protein (N) were found in testicular germ cells, endothelial cells in the heart, interdigitating cells in the thymus, dendritic cells in the spleen and Peyer's patches [19,21]. In experimentally infected gnotobiotic pigs, PRRSV antigens were found in bronchiolar epithelial cells, arteriolar endothelial cells, monocytes as well as interstitial, alveolar, and intravascular macrophages using an immunogold-silver immunohistochemical staining [22]. PRRSV RNAs and antigens were also found in bronchiolar epithelial cells [23], epithelium-like cells of alveolar ducts [24], and pneumocytes [23,25] in the naturally infected pigs whereas they were not found in these types of cells in the experimentally infected pigs [26]. Tissues such as lung, lymphoid tissues, Peyer’s patches, and kidney were also the preferable target organs of PRRSV infection [27,28].

*In vitro*, PRRSV was first isolated on primary cultures of PAMs [4] and so far, these cells as well as freshly isolated blood monocytes or monocytic derived dendritic cells [29-31], remain the only non-genetically modified porcine cells that can be used for viral propagation since they can be infected by the virus and allow its replication. On the other hand, using primary cell lines present some disadvantages as low number of cells harvested, heterogeneity of the population, and more importantly short lifespan of cells. Thus, using *in vitro* cell lines present some benefits compared to primary cell lines. There are two non-porcine permissive immortalized cell lines that permit the complete replication cycle of PRRSV, the MARC-145 and CL2621 cells (subclones of MA104 monkey kidney cell line) [7,32,33] which are routinely used for *in vitro* propagation of PRRSV and for large scale production of PRRSV vaccine strains. More recently, new cell lines have been genetically modified to become permissive to PRRSV, as immortalized PAM cells expressing the CD163 protein [34], immortalized porcine monomyeloid cells expressing the human telomerase reverse transcriptase [35], PK-15 cells expressing the sialoadhesin protein [36], and porcine, feline and baby hamster kidney cells expressing the CD163 protein [37]. Thus, all new reported cell lines have been genetically modified to be permissive to PRRSV, leaving room for the discovery of non-genetically modified PRRSV permissive cell lines.

PRRSV can be airborne transmitted through long distance [38]. Airborne transmitted pathogens need to interact with host cells of the respiratory tract such as epithelial cells and alveolar macrophages in order to be able to enter and disseminate in the host organism. If PRRSV is airborne transmitted and PRRSV antigens and viral RNA can be detected in epithelial cells of the respiratory tract of infected pigs, then it can be speculated that, in addition to the alveolar macrophages, epithelial cells of respiratory tract could be permissive to PRRSV replication *in vitro*. Nonetheless, no immortalized epithelial cell of the respiratory tract of swine had been reported so far to be permissive to PRRSV infection and replication *in vitro* and attempts to find such cells have previously failed [4,39,40].

Thus, St-Jude porcine lung cells (SJPL) cells, which were at first reported to be an immortalized epithelial cells line of the respiratory tract of swine and were previously described to be suitable for influenza virus replication [41], were tested for their PRRSV permissivity. Noteworthy, during the course of this study, the SJPL cell line was found to be of monkey origin based on karyotype and genetic analyses [42]. Nevertheless, the results of the present study show that SJPL cells are: 1) permissive to PRRSV replication and 2) phenotypically different from MARC-145 cells.

## Results

### SJPL cells susceptibility to PRRSV

**Figure 1:**
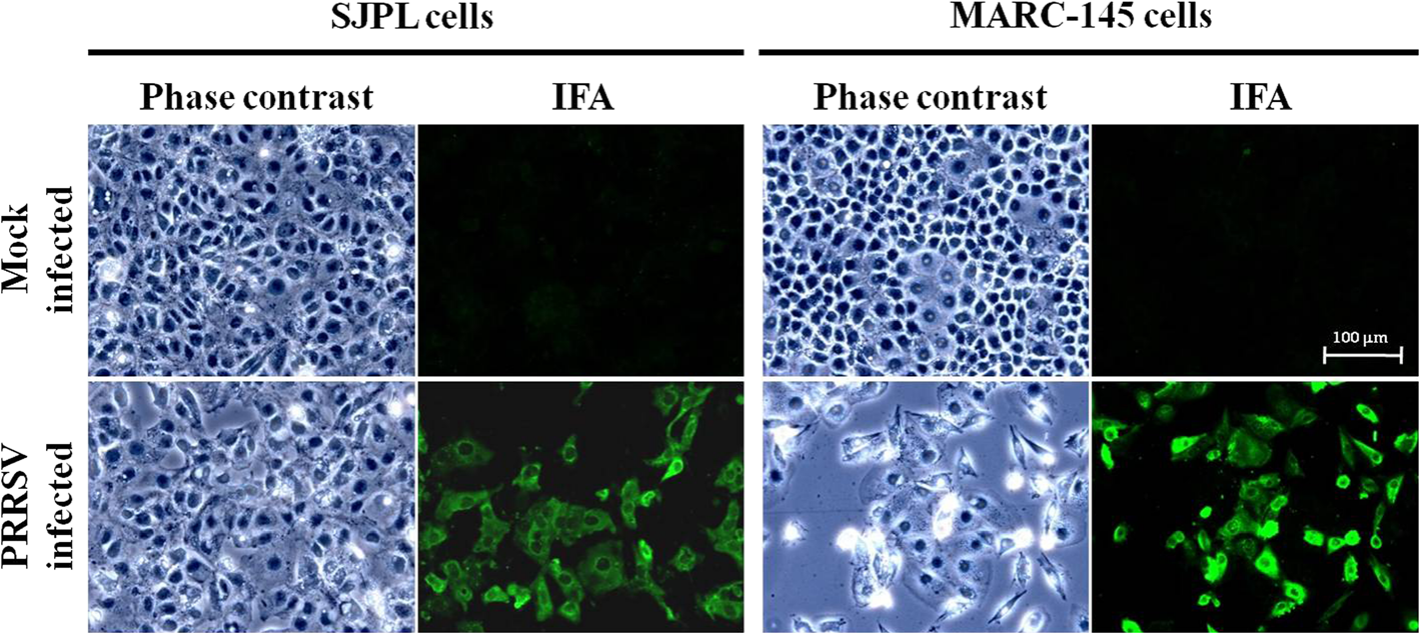
**Immunofluorescence detection of the N viral protein in PRRSV-infected SJPL cells.** The IFA was done at 72 hrs pi as described in the materials and methods section. Mock infected cells are illustrated as control in the upper panels. Cells infected at 1 MOI with PRRSV IAF-Klop reference strain are illustrated in lower panels. Cells were visualized with visible light (phase contrast) and UV (IFA). Arrows indicate the SJPL cell monolayer disruption induced by PRRSV infection.

In order to evaluate the susceptibility of epithelial cells of the respiratory tract of swine in regards to PRRSV, two epithelial cell lines, the NPTr and SJPL cells, were inoculated with PRRSV IAF-Klop strain at 1 multiplicity of infection (MOI). As reported previously, the NPTr cells were not permissive to PRRSV (data not shown) [40]. However, the SJPL cells infected by PRRSV developed a very light cytopathic effect (CPE) at 72 hrs post-infection (pi) compared to mock infected cells as illustrated in Figure 1, which suggested the replication of PRRSV. The amount of CPE observed in SJPL infected cells increased over time but it has always been significantly lower compared to PRRSV-infected MARC-145 cells (data not shown and Figure 1). The degree of CPE at 120 hrs pi in PRRSV-infected SJPL cells was similar to the amount of CPE observed at 72 hrs pi in PRRSV-infected MARC-145 cells (data not shown). Interestingly, the SJPL cells growth and cell dimension were higher (doubling time: 32.57 ± 2.58 hrs, surface: 4684.41 ± 2188.94 μm^2^, respectively) compared to MARC-145 cells (doubling time: 21.67 ± 3.30 hrs, surface: 3568.96 ± 1128.47 μm^2^, respectively) (Additional file 1: Figure S1). To confirm the PRRSV proteins expression in SJPL infected cells, an immunofluorescent assay (IFA) was performed. The PRRSV N protein was detected in PRRSV-infected SJPL cells (Figure 1) which indicates that PRRSV was able to express at least the N viral protein. Most of the IFA positive cells have positive signal localized in the cytoplasm (Figure 1) such as what has been reported previously for PRRSV-infected MARC-145 cells [43].

### Identification of PRRSV receptors in SJPL cells

**Figure 2:**
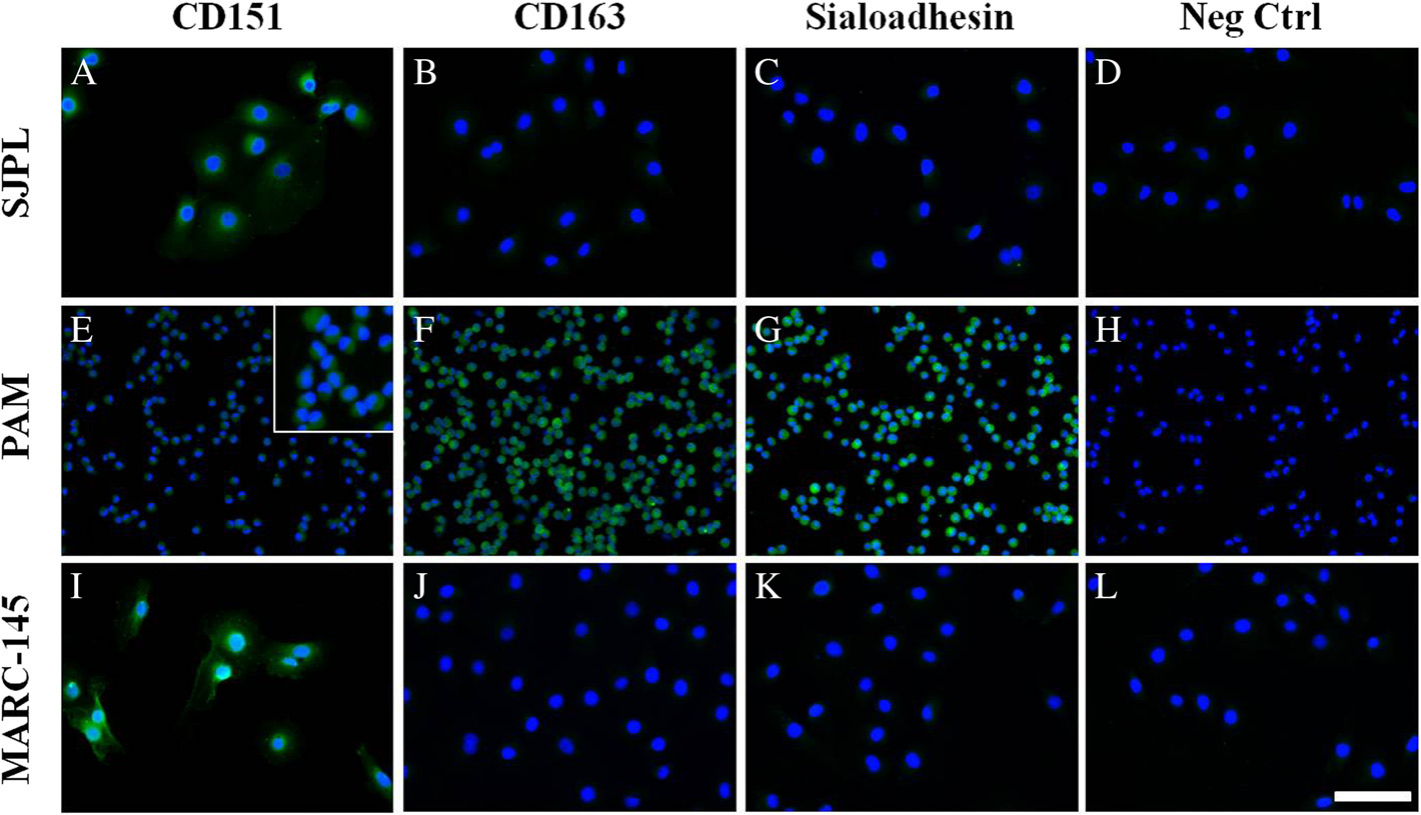
**Detection of PRRSV receptors in SJPL cell line by immunofluorescence.** The IFA was done using specific antibodies directed against CD151, CD163 and Sialoadhesin proteins in SJPL, PAM and MARC-145 cells. Negative control was obtained without primary antibody exposition. As an example of results, the negative controls illustrated are the FITC conjugated anti-mouse. All cell nuclei were stained with DAPI. Cells were visualized under UV exposition. Scale bar = 100 μm.

Three cellular molecules have been identified to play a critical role in rendering non-permissive cells susceptible to PRRSV infection: the CD163, CD151 and sialoadhesin (Sn) [44-47]. Thus, the presence of these molecules in SJPL cells was determined by an immunofluorescent assay (Figure 2). As illustrated in Figure 2A, the SJPL cells express the CD151 such as MARC-145 cells (Figure 2I) and to a lower extent such as the PAM cells (Figure 2E). The expression of CD163 and Sn proteins was detected only in PAM cells (Figure 2F and 2G).

### Infectious viral particles production in PRRSV-infected SJPL cells

**Figure 3:**
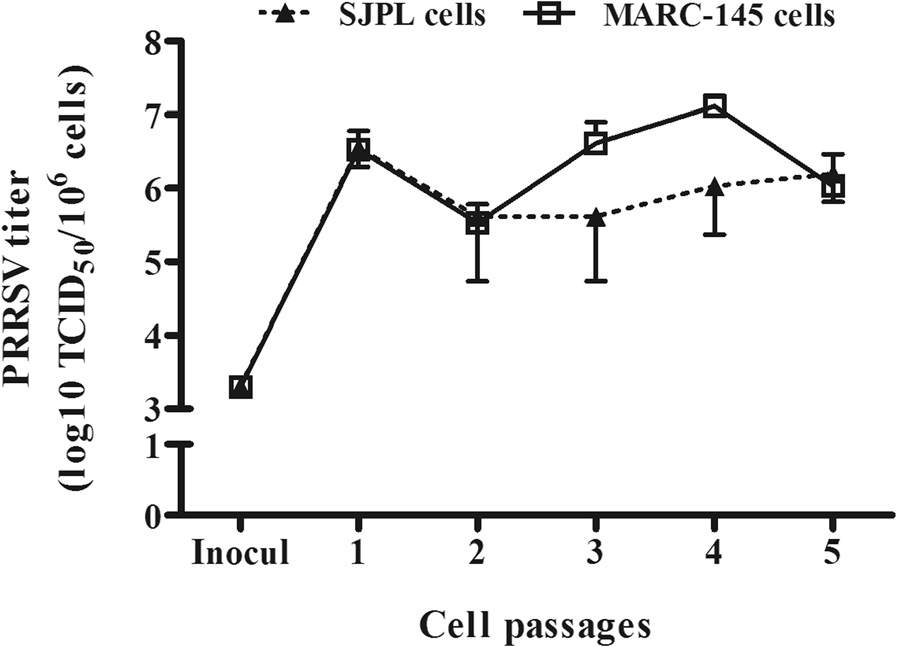
**Infectious viral particles production in PRRSV-infected SJPL cells following five consecutive passages.** PRRSV IAF-Klop strain was passaged serially in MARC-145 and SJPL cells as described in the materials and methods section. The amount of the infectious viral particles recovered after each passage was determined in MARC-145 cells. The virus titers were expressed as TCID_50_ per 10^6^ cells. The initial viral inoculum (inocul) used to infect both cell lines was 10^3.3^ TCID_50_/10^6^cells.

To establish if SJPL cells allow full PRRSV replication cycle and infectious particles production after being in contact with infectious virions, the amount of infectious PRRSV particles produced by SJPL cells was evaluated during five consecutive passages. As illustrated in Figure 3, the amount of infectious virus yield from the inoculum (10^3.3^ TCID_50_/10^6^ cells) compared to the first passage in SJPL cells (10^6.6^ TCID_50_/10^6^ cells) increased around 2000 times which indicates clearly that SJPL permits the production of infectious viral particles. The amount of virus yield was maintained during subsequent passages which further indicates that infectious PRRSV particles are produced (Figure 3). However, the overall production of infectious particles in SJPL cells compared to MARC-145 cells does not seem to be significantly different (*P *> 0.05).

**Figure 4:**
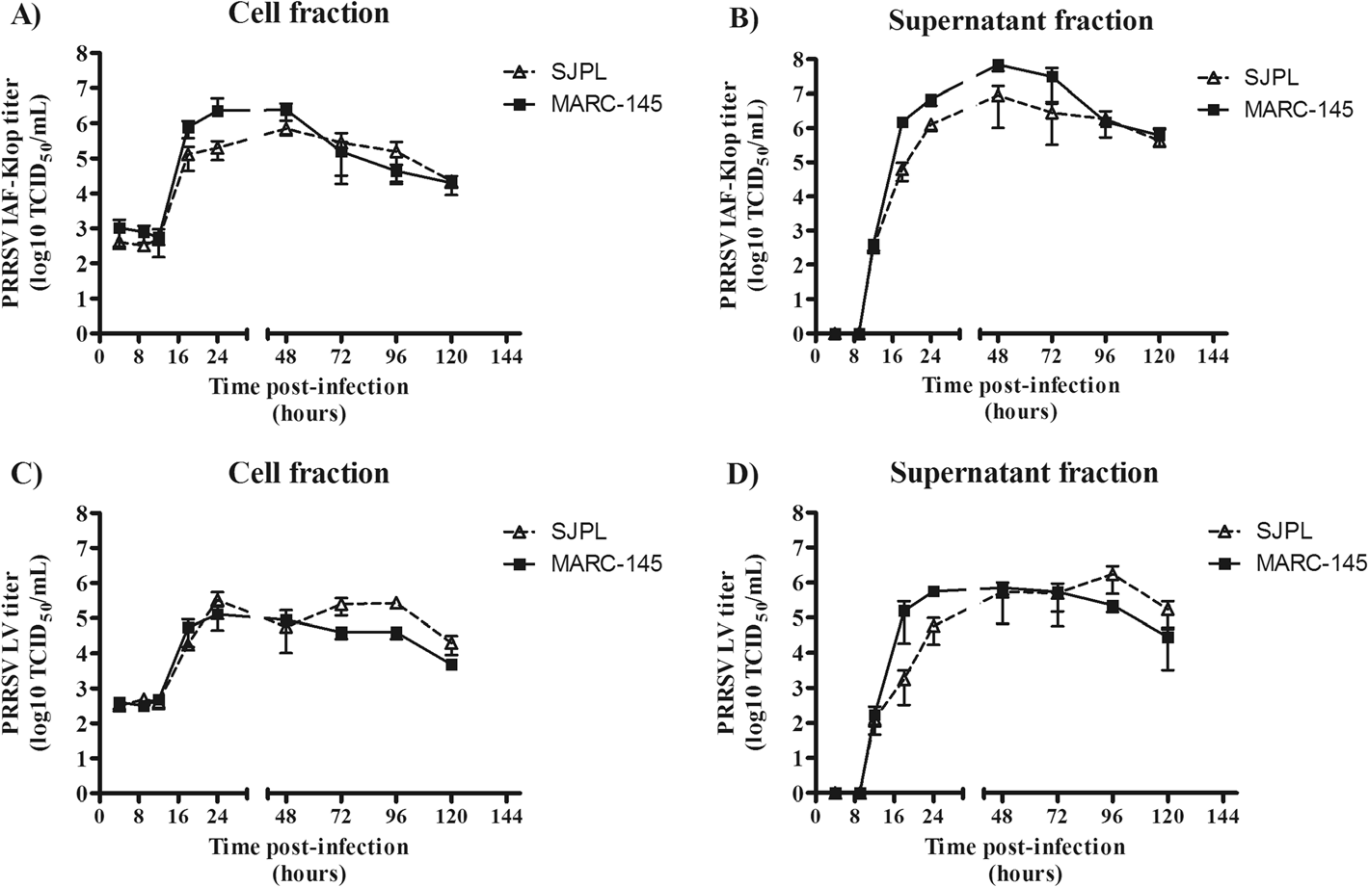
**PRRSV replication kinetics in SJPL cells.** MARC-145 and SJPL cells were infected at 1 MOI with PRRSV IAF-Klop (**A**, **B**) and LV (**C**, **D**) strains. At different time pi, the infectious viruses recovered from the cell culture medium (**B**, **D**: supernatant fraction) and the cells (**A**, **C**: cell fraction) were titered in MARC-145 cells. Experiment was done in triplicate.

In order to determine the efficiency of PRRSV production in SJPL cells compared to MARC-145 cells, a PRRSV replication kinetic experiment using type I and type II PRRSV reference strains (LV and IAF-Klop, respectively) has been conducted (Figure 4). Three significant dual interactions of fixed-effect variables were recorded: cell*matrix (*P *= 0.0224), cell*time (*P *= 0.0006), and matrix*time (*P *< 0.0001) (see methods section for the definition). Thus, as expected, the increase of the PRRSV titers over time was statistically significant for both cell types and both matrix (cell fractions and supernatant fractions). The viral titers of the SJPL cell fraction were 0.22 ± 0.08 TCID_50_ units (log10) lower than the ones of MARC-145 cell fraction (*P *= 0.0806) (Figure 4A), but those of the SJPL supernatants were 0.51 ± 0.08 TCID_50_ units (log10) lower than the ones of MARC-145 supernatants (*P *< 0.01) (Figure 4B). In general, around 10 times more infectious viruses have been collected from the supernatant fraction compared to the cell fraction (*P *≤ 0.0002) (Figure 4A and 4C compared to 4B and 4D, respectively). Moreover, the areas under the time-TCID_50_ curves (AUC) results indicated that the overall estimation of PRRSV infectious virions production of the SJPL cells averaged 98% of those of MARC-145 cells for cell fractions (Figure 4A) and 90% for supernatant fractions (Figure 4B).

### Virus isolation efficiency

Overall, 22 cases were tested. Three were PRRSV real-time PCR negative and used as controls. From the 19 PRRSV real-time PCR positive cases, 11 PRRSV isolates were obtained using MARC-145 cells compared to 8 with SJPL cells (Table 1). Consequently, the virus isolation efficiency with MARC-145 and SJPL cells were 58% and 42%, respectively, suggesting that MARC-145 could be slightly more suitable for PRRSV virus isolation from clinical samples. In addition, all virus isolation positive cases with SJPL cells were also virus isolation positive with MARC-145 cells. Interestingly, when the amount of PRRSV was higher than 500 TCID_50_ of PRRSV/gram of tissue, the virus isolation efficiency was also very high for both cell lines. More precisely, the virus isolation efficiency for tissues that have > 500 TCID_50_ of PRRSV/gram was 100% and 88% for MARC-145 and SJPL cells, respectively (Table 1). To further characterize the PRRSV strains that were isolated, the ORF5 gene of five cases that were both virus isolation positive with MARC-145 and SJPL cells were sequenced. Sequence analyses revealed that all PRRSV strains are type II isolates (data not shown). The nucleotide (nt) identities between the tissues and the fourth cell passage in both cell lines of each cases were 100% identical indicating that the same PRRSV strains, that were identified initially in the tissues, were isolated. Moreover, at the fourth cell passage, the ORF5 sequences of viruses isolated from each porcine tissue homogenate in SJPL and MARC-145 cells were 100% identical which suggests that SJPL cells allow the isolation of the same strains as those isolated with MARC-145 cells. Sequence analyses also revealed genetic variability between strains that were isolated from each porcine tissue homogenate with SJPL cells (86.4% to 93.2% nt identities) and compared to the PRRSV reference strain IAF-Klop (88.3% to 91.0% nt identities).

**Table 1:** PRRS virus isolation efficiency from swine samples using SJPL cells compared to MARC-145 cells

**Amount of PRRS virus in tissues**^**a**^	**Cell types**	
	**MARC-145**	**SJPL**
**(TCID**_**50**_**/g)**	**(number isolated/number tested)**^**b**^	**(number isolated/number tested)**
0-100^c^	0 / 7	0 / 7
101-500	2 / 6	0 / 6
501-2500	7 / 7	6 / 7
2501-40000	2 / 2	2 / 2
Total:	11 / 22	8 / 22

^a^The PRRSV was quantified as previously described using a real-time PCR assay [67].

^b^The virus isolation were attempted with both cell lines using the same swine tissue homogenates.

^c^Three cases out of 7 were real-time PCR negative for the presence of PRRSV.

### PRRSV-induced apoptosis in SJPL cells

**Figure 5:**
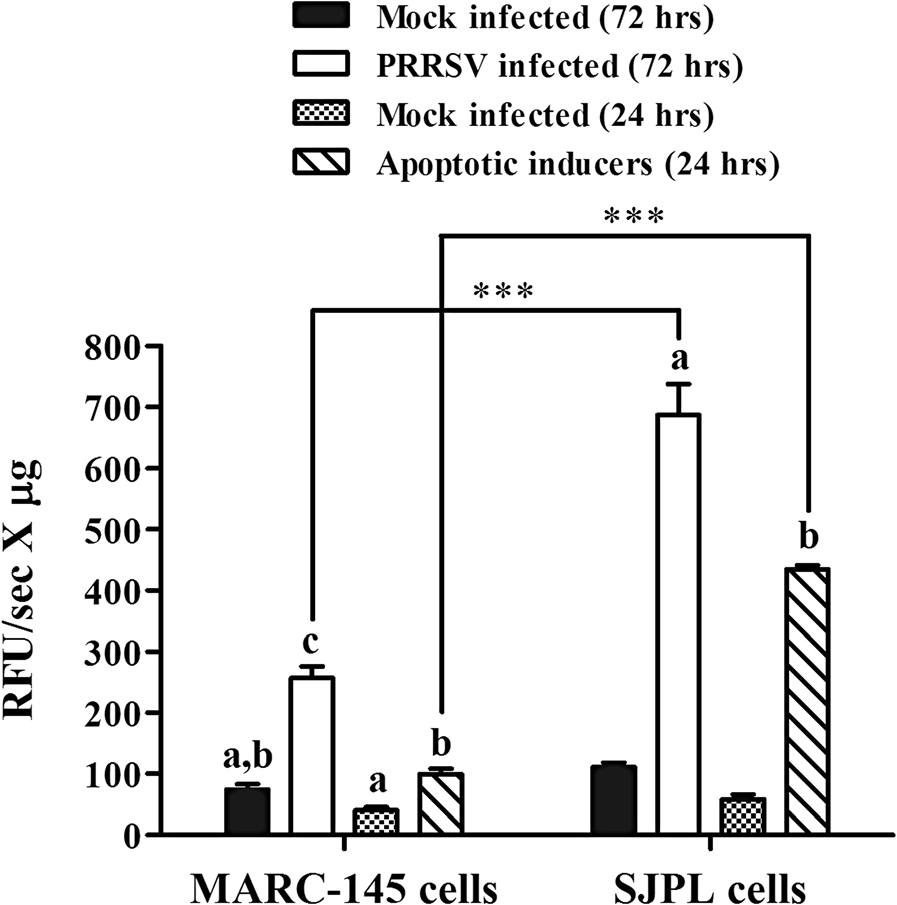
**Procaspases 3/7 activation in SJPL cells infected by PRRSV.** MARC-145 and SJPL cells were infected at 0.5 MOI with PRRSV IAF-Klop strain or incubated with a combination of four apoptotic inducers (actinomycin D, vinblastine sulfate, cycloheximide and puromycin) as a positive control. At 24 hrs post-incubation with the apoptotic inducers, MARC-145 cells have developed high CPE level compared to SJPL cells which showed low to mild CPE. At 72 hrs, PRRSV infected cells were disrupted for the detection of caspase 3 using a specific fluorogenic substrate. The results were expressed as relative fluorescence released (relative fluorescence units or RFU) per second per μg of cell lysates. Values are presented as ± standard deviation (SD). When 2 sets of data within a cell type are labeled with superscripts of different letters or when only one set is labeled with a superscript, it indicates that these 2 sets of data are statistically different (*P* < 0.05). When 2 sets of data from different cell types are labeled with asterisk, it indicates that these 2 sets of data are statistically different (*** *P* < 0.001).

As illustrated in Figure 5, the procaspases 3/7 activation by the IAF-Klop PRRSV reference strain and several apoptotic inducers was more pronounced in SJPL cells compared to MARC-145 cells (2.7 to 4.4 times higher). In addition, activation of procaspases 3/7 in PRRSV-infected MARC-145 and SJPL cells was 3.5 to 6.2 times higher (*P *< 0.05), respectively, compared to noninfected cells (Figure 5). Even if procaspases 3/7 activation was higher in SJPL cells, at the time the cells were disrupted (24 hrs and 72 hrs pi), the CPE was very mild in SJPL cells compared to MARC-145 cells (Figure 1). At least 60% of the MARC-145 infected cells showed CPE compared to SJPL cells which showed mild if no CPE.

### Inhibition of PRRSV replication

To evaluate the impact of IFNα in regards to PRRSV replication, different amounts of IFNα were added in the cell culture media of both cell lines. Interestingly, the minimal concentration of IFNα needed to have an antiviral effect against PRRSV replication was 8 to 16 times lower in SJPL cells (between 0.78 to 3.13 U/μL) compared to MARC-145 cells (between 6.25 to 50 U/μL) (Table 2), showing that SJPL cells are more sensitive to IFNα than MARC-145 cells.

**Table 2:** Minimal concentration of IFNα for the inhibition of PRRS virus infection

	**PRRSV infection inhibition**	
**Cells**	**Cytopathic effect inhibition**	**Immunofluorescence inhibition**
MARC-145	> 6.25, < 12.5^a^	> 50, < 78.13
SJPL	> 0.78, < 1.56	> 3.13, < 6.25

^a^Express in U/μL.

Note: the experiment was done in duplicate.

### Effect of PRRSV on cytokine mRNAs expression

**Figure 6:**
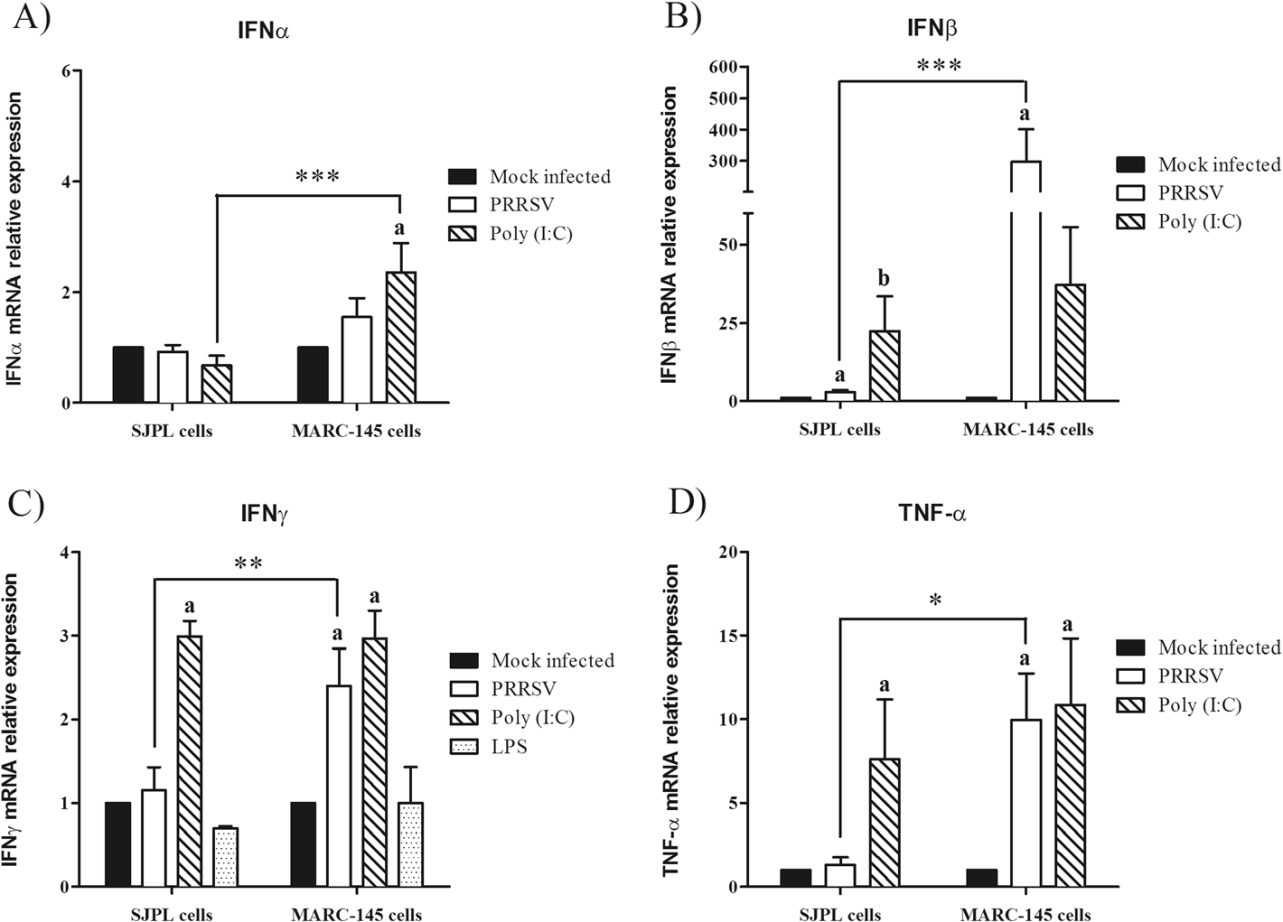
**Relative expression of IFNα (A), IFNβ (B), IFNγ (C) and TNF-α (D) mRNA of SJPL and MARC-145 cells induced by PRRSV.** MARC-145 and SJPL cells were infected at 0.5 MOI with PRRSV IAF-Klop strain or transfected with poly (I:C) as a positive control or treated with LPS as an IFNγ inducer. mRNA relative expression of IFNα (A), IFNβ (B), IFNγ (C) and TNF-α (D) was measure at 72 hrs by qRT-PCR in PRRSV infected or poly (I:C) treated cells. Values are presented as ± standard deviation (SD). When 2 sets of data within a cell type are labeled with superscripts of different letters or when only one set is labeled with a superscript, it indicates that these 2 sets of data are statistically different (*P* < 0.05). When 2 sets of data from different cell types are labeled with asterisk, it indicates that these 2 sets of data are statistically different (* *P* < 0.05, ** *P* < 0.01, *** *P* < 0.001).

Since the sensibility to IFNα PRRSV inhibition was different between SJPL and MARC-145 cells, the level of mRNA expression of different cytokines known to be regulated following different viral infections, such as IFNα, IFNβ, IFNγ, and TNF-α, [48], was evaluated. As previously described, PRRSV infection in MARC-145 or in PAM cells induced an increase of IFNβ, IFNγ and TNF-α, but not of IFNα mRNAs expression [49-53] (Figure 6). On the other hand, PRRSV infection only induced an increase expression of IFNβ, and not of the other tested cytokines mRNA in infected SJPL cells compared to MARC-145 cells, where PRRSV induced IFNβ, IFNγ, and TNF-α mRNAs. Transfecting SJPL and MARC-145 cells with Poly (I:C) as a positive control induced an increase of IFNβ, IFNγ and TNF-α mRNAs in both cell types but no increase of IFNα mRNA was observed. However, SJPL cells were found to have a basal level of expression of IFNα mRNA similar to MARC-145 cells (data not shown) suggesting their ability to produce the protein. Furthermore, Poly (I:C) increased the relative expression of IFNβ, IFNγ and TNF-α mRNAs in a similar way in both MARC-145 and SJPL cells, suggesting that both cell types can produce the tested mRNA cytokines. Interestingly, LPS was not able to induce IFNγ mRNA expression in both cell lines. Statistical analyses indicated that the relative expression of IFNβ, IFNγ and TNF-α mRNAs are significantly different between PRRSV-infected MARC-145 and SJPL cells, adding another evidence that the two cell lines are responding differently following PRRSV infection.

## Discussion

Previous attempts to find porcine immortalized cell lines, not genetically modified, into which PRRSV infectious particles could bind, enter and complete a full virus replication cycle including infectious virions production, such as epithelial cell line of the respiratory tract, have failed [4,39,40]. In the present study, a new immortalized cell line, the SJPL cells [41], was found to be permissive to PRRSV infection and replication (Figures 1, 3 and 4). At the beginning of this project, the SJPL cells were reported to be epithelial cells of the respiratory tract of swine [41,54]. During the course of this study however, these cells were found to be of monkey origin [42], suggesting that they could be phenotypically similar to MARC-145 cells, another monkey cell line. In addition, the SJPL cells have been known to be permissive to a variety of sub-types of influenza virus from human, swine, avian and horse origins [41]. From now on, PRRSV can be added to the list of viruses that can replicate in SJPL cells.

Although, the SJPL cells were found to be phenotypically distinguishable from the MARC-145 cells based on cell growth curve (Additional file 1: Figure S1), cell dimension (Figure 1), and CPE development following PRRSV infection (Figure 1), the amount of infectious virus produced in PRRSV-infected SJPL cells (Figure 4) was similar to PRRSV-infected MARC-145 indicating that those differences do not seem to affect the virus production. In addition, it is suggesting that SJPL could replace the MARC-145 cells (and related cells that derivate from MA104 cells) in a large scale PRRSV live or killed vaccine production. Interestingly, even if SJPL cells seem to be slightly less sensitive for PRRSV isolation compared to MARC-145 cells (Table 1), the SJPL cells were able to allow the replication of several PRRSV type II ORF5 genomic variants and the type I reference strain LV (Figure 4 and Table 1) indicating that at least these cells are permissive to a wide spectrum of PRRSV isolates.

The IFNα antiviral effect against PRRSV-infected MARC-145 cells has been previously reported [55]. Consequently, different amount of IFNα were added in the cell culture media to evaluate its antiviral effect in regards to both PRRSV-infected cell lines. It was found that SJPL cells are more responsive to the IFNα antiviral effect than MARC-145 cells (Table 2). The level of cytokine mRNA expressions measured by qRT-PCR was different between SJPL and MARC-145 cells. As previously demonstrated in other studies, PRRSV infection in MARC-145 or PAM cells induced an increase in IFNβ, IFNγ and TNF-α mRNA expressions [49-53] and similar results were obtained in this study. On the other hand, in SJPL cells, PRRSV infection only induced IFNβ mRNA expression at much lower amount compared to MARC-145 infected cells, indicating that PRRSV might escape IFN type I and other cytokines responses [31,56,57]. Several published reports showed that PRRSV contained an ability to suppress the IFNβ activity at the transcription level [49,58,59]. All those studies looked either at the promoter induction (Luciferase assay) or at the mRNA level by qRT-PCR at 0 hr until 48 hrs post-infection (pi). In the present study, IFNβ mRNA was quantified at a different time pi compared to previous studies, i.e. at 72 hrs pi, which could explain the difference that has been observed. In Genini and collaborators (2008), a strong induction of IFNβ mRNA was observed in PRRSV-infected PAM cells with a variation in time pi, i.e.:, no induction at 0 hr, induction at 3 hrs pi, no induction at 6 hrs pi and very strong induction at 9 and 12 hrs pi, illustrating that there is a variation in time of IFNβ mRNA level that does not seem to be constant and proportional [53]. Furthermore, Lee and collaborators (2004), have reported that different strains of PRRSV are able to induce distinctive interferon phenotypes *in vitro* indicating that the induction of cytokines mRNA can vary substantially between PRRSV strains [60]. In the present study, a different PRRSV strain (IAF-Klop) was used compared to previous reports which might explain the differences in cytokine mRNA phenotype that was observed. Thus, those results demonstrate the importance of using more than one *in vitro* model to study PRRSV replication cycle and pathogenesis.

Many studies have demonstrated that PRRSV induces apoptosis both *in vitro* and *in vivo*[21,61-64] and several techniques have been used to demonstrate that phenomenon, such as procaspase 3 activation in PRRSV IAF-Klop infected MARC-145 cells [65]. The CPE visualized by light microscopy in PRRSV-infected SJPL cells was very mild and was delayed over time compared to PRRSV-infected MARC-145 cells (Figure 1, data not shown, respectively). The increase in TNF-α mRNA obtained with qRT-PCR in infected MARC-145 cells and the absence of its up regulation in infected SJPL cells (Figure 6) could support the difference observed in CPE. Consequently, amount of caspase 3 in MARC-145 cells infected by PRRSV is expected to be higher compared to SJPL cells infected by PRRSV. Surprisingly, the opposite situation was observed indicating that SJPL cells are more suited for procaspases 3/7 activation than MARC-145 cells (Figure 5). This latest result demonstrates clearly that SJPL cells are phenotypically completely different from MARC-145 cells and that the level of procaspases 3/7 activation and TNF-α mRNA expression induced by PRRSV may not be related to the level of CPE that could be observed by light microscopy. In fact, other cell death mechanisms have been reported to occur in cells infected by PRRSV such as necrosis which could explain this difference observed between the cell lines [30,66].

## Conclusions

In conclusion, SJPL cells are phenotypically different from MARC-145 cells and they respond differently to PRRSV infection (Figures 1, 5, and 6, Tables 2, Additional file 1: Figure S1). SJPL cells have also been shown to represent a convenient *in vitro* model for the study of porcine bacterial pathogens [54]. Thus, studying the PRRSV-SJPL interactions should give us new insight in regards to the viral pathogenesis of PRRSV. In addition, SJPL cells could serve as a new *in vitro* model to study viral-bacterial interactions during mixed infections.

## Methods

### Cells and viruses

MARC-145 cells, which are a subclone of the African green monkey kidney MA104 cells that is highly permissive to PRRSV [33], were maintained as described previously [59]. The St. Jude porcine lung (SJPL) epithelial cell line was provided kindly by Dr R.G. Webster (St. Jude Children's Hospital, Memphis, TN, USA) [41]. During this study, karyotyping and genome sequence analyses of the SJPL cells revealed that their species origin was not porcine but was rather monkey [42]. The newborn pig trachea epithelial cell line (NPTr) was provided kindly by Dr. M. Ferrari (Instituto Zooprofilattico Sperimental, Brescia, Italy) [40]. The SJPL and NPTr cell lines were cultured in Dulbecco’s modified Eagle’s medium (DMEM) (Invitrogen Corporation, GibcoBRL, Burlington, ON, Canada) supplemented with 10% fetal bovine serum (FBS) (Wisent Inc, St-Bruno, QC, Canada), 1 mM sodium pyruvate, 2 mM L-glutamine, 1 μM MEM nonessential amino acids, 10 U/mL of penicillin, 10 μg/mL of streptomycin and 250 g/L antibiotic-antimycotic solution (Invitrogen Corporation, GibcoBRL) as described previously [40,41]. Pulmonary alveolar macrophages (PAMs) were used as a positive controls for the detection of PRRSV receptors. PAMs were harvested from lungs of 2 to 14 weeks old pigs. Pigs were sacrificed following ethic protocol 12-Rech-1640 approved by the Institutional ethic committee following the guidelines of the Canadian Council on Animal Care (CCAC). Briefly, an instillation of the lungs with PBS containing 10 units/mL penicillin, 10 μg/mL streptomycin and 100 mg/L gentamicin (Invitrogen Corporation, GibcoBRL) was realized. Then, PBS was collected and PAMs removed following low speed centrifugation. Cells were washed with medium DMEM complemented with 2 mM L-glutamine, 0,1 mM HEPES, 1 μM Non-essential amino acids (Invitrogen Corporation, GibcoBRL), 250 g/L Amphotericin B (Wisent Inc), 10 units/mL penicillin, 10 μg/mL streptomycin and 100 mg/L gentamicin. Cells were then collected following low speed centrifugation and were resuspended in freezing medium (same as wash medium plus 20% fetal bovine serum (Wisent Inc.) and 10% DMSO (Sigma, St-Louis, MO, USA)) and slowly frozen, than stored in liquid nitrogen until further utilization. PAMs were cultured for 24 hours in complete DMEM prior the immunofluorescence assay. All cell lines were cultured at 37°C in 5% CO_2_ atmosphere.

The PRRSV strain used to establish the permissivity of the SJPL cells was the MARC-145 cells adapted IAF-Klop North American reference strain [65] and the Lelystad (LV) European reference strain [23]. The PRRSV virus stocks were obtained following three cycles of freeze-thaw of PRRSV MARC-145 infected cells. Afterward, the virus was purified following a 3.5 hrs period of ultracentrifugation on a 30% sucrose cushion (in a TBS solution: 50mM tris pH7.5, 150mM NaCl) using the SW28 Beckman Coulter rotor at 83,000 relative centrifugal force (rcf). The virus pellets were resuspended in 0.5 mL of PBS and aliquots of the virus stocks were then conserved at –70°C for future use. The infectious dose of the virus stocks was calculated from a 96-well microplate of MARC-145 infected cells by the Kärber method as described previously [67]. Virus titers were expressed in tissue culture infectious dose 50% per mL (TCID_50_/mL).

### Immunofluorescence assay (IFA) for the detection of PRRSV antigen

The presence of PRRSV antigens in infected cells was determined by an immunofluorescence assay (IFA). Briefly, cells infected by PRRSV strains were fixed at different times post-infection (pi) with a 4% paraformaldehyde (PFA) solution prepared as described previously [68]. Mock-infected cells were included as negative controls. After an incubation period of 30 minutes at room temperature, the PFA solution was removed and cells were washed three times with a phosphate buffer saline solution (PBS). Then, cells were incubated during 10 minutes at room temperature with a PBS solution containing 1% Triton X-100. After removing the Triton X-100 solution, the cells were washed three times with a PBS-Tween 20 solution (PBS containing 0.02% Tween 20). After the permeabilization procedure, cells were incubated 30 minutes with PBS containing 0.2% Tween 20 and 1% Fetal Bovine Serum Albumin. Then, the α7 rabbit monospecific antisera (a specific anti-N PRRSV protein antibody) [65] was diluted 1/200 in the washing buffer and added to the cells and incubated at room temperature for a 30 minutes period. Cells were then washed and incubated for 30 minutes with the washing buffer containing a 1/160 dilution of anti-rabbit specific antisera FITC conjugated (Sigma-Aldrich Inc., St-Louis, USA). Finally, cells were visualized using a DMI 4000B reverse fluorescence microscope, image of the cells were taking with a DFC 490 digital camera and the image were analyzed using the Leica Application Suite Software, version 2.4.0 (Leica Microsystems Inc., Richmond Hill, Canada).

### Immunofluorescence assay for PRRSV receptors CD151, CD163, and Sialoadhesin detection

The presence of CD151, CD163 and Sialoadhesine (Sn) proteins in MARC-145, SJPL and PAM cells was determined by an IFA. Briefly, cells were fixed with a 4% PFA solution as described previously [68]. After an incubation period of 30 minutes at room temperature, the PFA solution was removed and cells were washed three times with PBS. Then, cells were incubated with a permeabilization and blocking solution, PBS solution containing 0.1% Triton X-100, 7% normal sheep serum (NSS) and 5% non fat dry milk (NFDM), during 30 minutes at room temperature. After removing the permeabilization/blocking solution, the cells were washed three times with a PBS. Then, cells were incubated overnight with primary antibodies. All those antibodies were diluted 1/100 in antibody solution containing PBS, 0.1% Triton X-100, 1.4% NSS, and 1% NFDM. The antibodies used were: rabbit polyclonal anti-human CD151 (Santa Cruz Biotechnology, CA, USA); mouse monoclonal anti-pig CD163 (AbD Serotec, Oxford, United Kingdom) for PAMs cells or goat polyclonal anti-human CD163 (Santa Cruz Biotechnology, CA, USA) for MARC-145 and SJPL cells; mouse monoclonal anti-pig CD169 (synonym: Sialoadhesin, Siglec-1) (AbD Serotec, Oxford, United Kingdom) for PAMs cells or goat polyclonal anti-human Siglec-1 (Santa Cruz Biotechnology, CA, USA) for MARC-145 and SJPL cells. The anti-CD163 and anti-CD169 polyclonal antibodies are known to react against several animal species. Their reactivity was confirmed against the PAMs control positive cells (data not shown). Thereafter, cells were washed three times and incubated for 60 minutes with the antibody buffer containing a 1/160 dilution of anti-rabbit specific antisera FITC conjugated (Sigma-Aldrich Inc., St-Louis, USA) or 1/200 dilution of anti-mouse specific antisera FITC conjugated (ICN Immuno Biological, CA, USA). Nuclei were stained with 4',6-diamidino-2-phenylindole DAPI (Sigma-Aldrich Inc., St-Louis, USA) as recommended by the manufacturer. Negative controls were obtained from cells where only the primary antibody was omitted. Cells were visualized the same way as described above.

### Virus production during multiple cell passages

An amount of 10^6^ MARC-145 or SJPL cells were infected with 0.005 MOI of IAF-Klop strain. Then, cells with their supernatants were subjected to three cycles of freeze-thaw at -70°C and the virus stock solutions were kept at -70°C for future use. Four subsequent viral passages in MARC-145 and SJPL cells were done as described above except that a dilution of 1/20 of the previous viral stock solutions was used for cell infection. Mock-infected cells were included as controls in each passage. The amount of virus production at each passage was calculated from a 96-well microplate of MARC-145 infected cells by the Kärber method and the results were expressed in tissue culture infectious dose 50% per 10^6^ infected cells (TCID_50_/10^6^ cells).

### Virus replication kinetics assay

10^5^ MARC-145 and SJPL cells were infected with IAF-Klop and LV strains using an MOI of 1. At different times pi (0, 4, 9, 12, 18, 24, 48, 72, 96 and 120 hrs), whole cell cultures were collected and were processed by low speed centrifugation to separate the cell pellet (cell fraction) from the culture medium (supernatant fraction). Both fractions were stored at -70°C until used. Afterwards, virus titration was performed in MARC-145 cells as described above. Mock-infected cells were included in each experiment as controls. All experiments were repeated two times in triplicate.

### Virus isolation

Virus isolation was attempted from 22 swine samples (lung and lymph nodes tissues) submitted from October 2007 to September 2008 to the Veterinary virology diagnostic laboratory of the Veterinary College of the Université de Montréal. Those samples originated from 3 to 10 weeks old animals housed in different Canadian farms and they were submitted for different reasons such as PRRSV outbreaks, porcine circovirus associated disease outbreaks, or others health problems. Three of the submitted samples were PRRSV negative by a real-time PCR diagnostic assay (Tetracore Inc., Rockville, MD, USA) and the amount of infectious PRRSV contained in the 19 real-time PCR positives cases was determined using the same assay as described previously [67]. For virus isolation, about 1-2 cm^3^ of pool of tissue samples were homogenized and resuspended in 9 mL of culture medium without FBS. Then, three cycles of freeze-thaw at -70°C were performed and tissues homogenates were centrifuged and the supernatants were filtered (with a filter size of 0.2 micron). Following the sample treatment, 1 mL of filtered sample was used to inoculate cells and cells were incubated for 5 days. Then, three cycles of freeze-thaw were performed at -70°C and cell lysates were centrifuged at 4000 rpm for 10 min. Supernatants of cell lysates were collected and used for a subsequent cell infection cycle. One mL of the cell lysate supernatants was inoculated to freshly prepared cell cultures and cells were incubated for 5 days. This new infection step was done for three consecutive times. At the fourth passage, the virus isolation status was confirmed by the presence of CPE and a positive IFA result. To further characterize the PRRSV strains that were isolated from both cell lines, PCR products encompassing the ORF5 gene were obtained from tissues and fourth virus isolation cell passages, and sequenced subsequently. Sequences were analyzed using the CLUSTAL W alignment method of the BioEdit sequence alignment editor version 7.0.9 software (Ibis Therapeutics, Carlsbad, CA, USA).

### Apoptosis

MARC-145 and SJPL cells were infected with PRRSV IAF-Klop strain at 0.5 MOI or were incubated with a mix of apoptotic inducers (500 μg/mL actinomycin D, 60 nM vinblastine sulfate, 100 μg/mL cycloheximide and 40 μg/mL puromycin 2HCl; Biomol Research Laboratories Inc., Plymouth meeting, PA, USA) as positive controls. Cellular changes associated with the infection or the inducers were visualized respectively at 72 hrs pi and 24 hrs post-incubation, respectively, under a light microscope (Leica Microsystems Inc.). At this time, cells were disrupted in a lysis buffer (50 mM HEPES, pH 7.4, 100 mM NaCl, 0.1% CHAPS, 1 mM DTT and 100 μM EDTA) for 5 minutes followed by sonication (Sonifier S-450A, Branson, Danbury, CT, USA). Then, protein concentrations were measured by a Bradford assay following the manufacturer’s instructions (Bio-Rad Laboratories Ltd, Mississauga, ON, Canada). Subsequently, apoptosis was assessed by detecting the activation of procaspases 3/7 as described by Gagnon *et al.* (2003), with minor modifications [65]. Briefly, a volume of cell lysate corresponding to 50 μg of total cell protein was added to the assay buffer (50 mM HEPES, pH 7.4, 100 mM NaCl, 0.1% CHAPS, 1 mM DTT, 100 μM EDTA and 10% glycerol). Then, specific substrate for caspases 3/7, the Ac-DEVD-AFC fluorogenic substrate (Biomol Research Laboratories Inc.), was added at a final concentration of 200 μM and the rate of fluorescence released was monitored with a 96-well plate fluorometer (Synergy HT, Biotek, Winooski, VT, USA). The results were expressed as relative fluorescence released (relative fluorescence units or RFU) per second per μg of cell lysates.

### Inhibition of PRRSV replication

To determine the amount of porcine IFNα that is able to inhibit the replication of PRRSV in permissive cell lines, 10^4^ MARC-145 and SJPL cells were incubated overnight. The cells were then infected with the PRRSV IAF-Klop strain at an infectious dose of 0.5 MOI in a culture medium without FBS and incubated during 4 hrs. The culture medium was then removed and replaced by a complete medium (i.e. with 10% FBS) with different serially diluted concentrations of porcine IFNα (PBL, New Jersey, USA) and incubated during 5 days. Then, the development of CPE was monitored and an IFA was performed. All the experiments were done in duplicate.

### Analysis of cytokine mRNAs expression by real time reverse transcriptase-quantitative PCR

SJPL and MARC-145 cells were infected as described above or transfected with Polyinosinic–polycytidylic acid potassium salt (Poly (I:C) [50 μg/mL] (Sigma-Aldrich Inc., St-Louis, USA) as a positive control for innate immunity induction, using polyethylenimine (PEI) [1 μg/μL] (Sigma-Aldrich Inc., St-Louis, USA), for 72 hours or treated with 1μg/ml of lipopolysaccharide (LPS) (Sigma-Aldrich Inc., St-Louis, USA) for 20 hours, as an IFNγ inducer. Total cellular RNA was extracted from cells using Trizol reagent (Invitrogen, Burlington, ON, Canada) according to the manufacturer’s protocol. Quantification of RNA was performed with a Nanodrop (NanoDrop Technologies, Inc., Wilmington, Delaware, USA). 1 μg of total RNA was reverse-transcribed using the QuantiTect reverse transcription kit (Qiagen, Mississauga, ON, Canada). The cDNA was amplified using the SsoFast™ EvaGreen® Supermix kit (Bio-rad, Hercules, CA, USA). The PCR amplification program for all cDNA consisted of an enzyme activation step of 3 min at 98°C, followed by 40 cycles of a denaturing step for 2 sec at 98°C and an annealing/extension step for 5 sec at 58°C. The primers used for amplification of the different target cDNA are for IFNα: F-GCCCTTTGCTTTACTGATGG; R-TCTGCTCATTTGTTTCAGGAG, IFNβ: F-TCCTGTGGCAATTGAATGG; R-AATAGCGAAGATGTTCTGGAG, IFNγ: F-ACTCGAATGTCCAACGCAAAGCAG; R-TCGACCTCGAAACATCTGACTCCT, TNF-α: F-TCTGTCTGCTGCACTTTGGAGTGA; R-TTGAGGGTTTGCTACAACATGGGC. All primers were tested to achieve amplification efficiency between 90% and 110%. The primer sequences were all designed from the NCBI GenBank mRNA sequences using web-based software primerquest from Integrated DNA technologies [69]. The Bio-Rad CFX-96 sequence detector apparatus was used for the cDNA amplification. The quantification of differences between the different groups was calculated using the 2^-ΔΔCt^ method. Beta-2 microglobulin (B2M) was used as the normalizing gene to compensate for potential differences in cDNA amounts. The B2M primers from monkey origin used are F-GTGCTATCTCCACGTTTGAG and R-GCTTCGAGTGCAAGAGATTG. The non-infected MARC-145 and SJPL cells were used as the calibrator reference in the analysis.

### Statistical analyses

A two-way ANOVA model, followed by Bonferroni *post-hoc* tests (Graphpad PRISM Version 5.0 software) were used to determine if a statistically significant difference exists between MARC-145 and SJPL cell lines in regards to the amount of PRRSV produced after multiple cell passages, procaspases 3/7 activation and cytokines mRNA up regulation in PRRSV infected cells and in Poly (I:C) transfected cells. One-way ANOVA model, followed by Tukey's Multiple Comparison Test (Graphpad PRISM) were used to determine if a statistically significant difference exists between treatments within MARC-145 or SJPL cells in procaspases 3/7 activation and their mRNA relative expression of IFNα, IFNβ, IFNγ, and TNF-α. Differences were considered statistically significant with a p<0.05. For the viral replication kinetic experiment, the time-course of TCID_50_ measured from the cell pellets and supernatants was analyzed with SAS Version 9.1 software. The following linear mixed-effect model for repeated measurements was solved using restricted maximum-likelihood estimation [70]: *Y*_*ijkl*_ = *μ* + *α*_*i*_ + *β*_*j*_ + *τ*_*k*_ + (*α·β*)_*ij*_ + (*α·τ*)_*ik*_ + (*β·τ*)_*jk*_ + (α·β·*τ*)_*ijk*_ + *R·*(*α·β*)_*ijl*_ + *e*_*ijkl*_. Where *Y*_*ijkl*_ is the measured TCID_50_; *μ* is the grand mean; cell line (*α*_i_), type of analytical matrix (*β*_*j*_), and sampling time (*τ*_*k*_) are fixed factors; the experiment replicate (*R*_*l*_) is a random effect; and *e*_*ijkl*_ is the random error term. As indicated in the equation above, this statistical model included all dual and triple interactions between the fixed-effect factors, and the random-effect factor *R*_*l*_ was nested within cell and analytical matrix. The strategy for covariance structure modeling proposed by Littell et al (2000) was used [71]. Briefly, the model was estimated first with a free covariance structure. After inspecting the estimated covariance matrix, the model was estimated anew with more parsimonious covariance models (e.g., compound symmetry, first-order autoregressive), which structure resembled that of the unstructured covariance matrix. The heterogeneous first-order autoregressive covariance model was selected because it was the best fit to the empirical covariance matrix, as determined with the Akaike information criterion [71]. Least-square means were used to assess differences between the two cell lines at each time and for each type of analytical matrix (i.e., cell or supernatant fractions), using Bonferroni-adjusted significance thresholds. The areas under the time-TCID_50_ curves (AUC) were calculated for each cell*matrix*replicate in order to obtain estimates of total viral production for each cell line following the 120 hrs duration of the experiment.

## Abbreviations

AUC: Areas under the time-TCID_50_ curves; B2M: Beta-2 microglobulin; CPE: Cytopathic effect; EAV: Equine arteritis virus; EU: European genotype; IFA: Immunofluorescence assay; IFN: Interferon; LDV: Lactate dehydrogenase-elevating virus; LPS: Lipopolysaccharide; LV: Lelystad virus; N: Nucleocapsid protein; NA: North American genotype; NPTr: Newborn trachea epithelial cell line; ORF: Open reading frame; PAM: Porcine alveolar macrophages; PBS: Phosphate buffer saline solution; PCV: Porcine circovirus; PCV2: Porcine circovirus type 2; PEI: Polyethylenimine; PFA: Paraformaldehyde; Pi: Post-infection; Poly (I:C): Polyinosinic–polycytidylic acid potassium salt; PRRS: Porcine reproductive and respiratory syndrome; PRRSV: Porcine reproductive and respiratory syndrome virus; Rcf: Relative centrifugal force; SJPL: St-Jude porcine lung cells; TCID_50_: 50% tissue culture infective dose; TNF-α: Tumor necrosis factor alpha.

## Competing interests

The authors declare that they have no competing interests.

## Authors’ contributions

CP carried the qRT-PCR studies and their statistical analyses, the PRRSV cell receptors immunofluorescence assay, the PAM cells harvesting, the cell growth curves and participated in the writing of the manuscript. JJJ carried out the PRRSV multiple cell passages production, some of the kinetic studies, and participated in the writing of the manuscript. CP and JJJ have contributed equally to this manuscript. CL carried out the IFNα inhibition studies, and participated in the writing of the manuscript. NM did the microscopy studies (SJPL permissivity), the virus isolation efficiency, some of the kinetic studies, and participated in the writing of the manuscript. MEL carried the apoptosis studies. JREDC performed the statistical analyses of the virus replication efficiency. MJ and CAG conceived the study, realized its design, supervised the graduate students and helped to draft the manuscript. In addition, CAG conducted the *in vivo* experiment for the harvesting of PAM cells. All authors read and approved the final manuscript.

## Supplementary Material

Additional file 1 **Figure S1.** SJPL and MARC-145 cells growth curves. Amount of cells (log2) was calculated with a hemacytometer at seeding time (0 hr), and at 24, 48, and 72 hrs post-incubation. Doubling time was calculated with data obtained at 24 and 48 hrs post-incubation with the formula: Doubling time (hrs) = 0.3 X incubation time (hrs) / (log cell time b- log cell time a), where incubation time is 24 hrs, cell time a is the amount of cells at 24 hrs and cell time b is the amount of cells at 48 hrs. Interestingly, the MARC-145 cells grow faster than the SJPL cells. During this experiment, cells surface areas were calculated with ImageJ v.1.6.0 from three different pictures of each cell type at 24 hrs post-incubation. SJPL and MARC-145 cells have a surface area of 4684.41 ± 2188.94 μm^2^ (n = 29) and 3568.96 ± 1128.47 μm^2^ (n = 38), respectively. Statistical analyses using t test showed that SJPL cells have a statistically wider surface than MARC-145 cells (*P *< 0.01) (data not shown). Click here for file
