# Supplementary figures and images for: Identification of a new cell line permissive to porcine reproductive and respiratory syndrome virus infection and replication which is phenotypically distinct from MARC-145 cell line
Source: Virol J. 2012 Nov 13;9:267. doi: 10.1186/1743-422X-9-267 (PMC3546013; doi:10.1186/1743-422X-9-267)

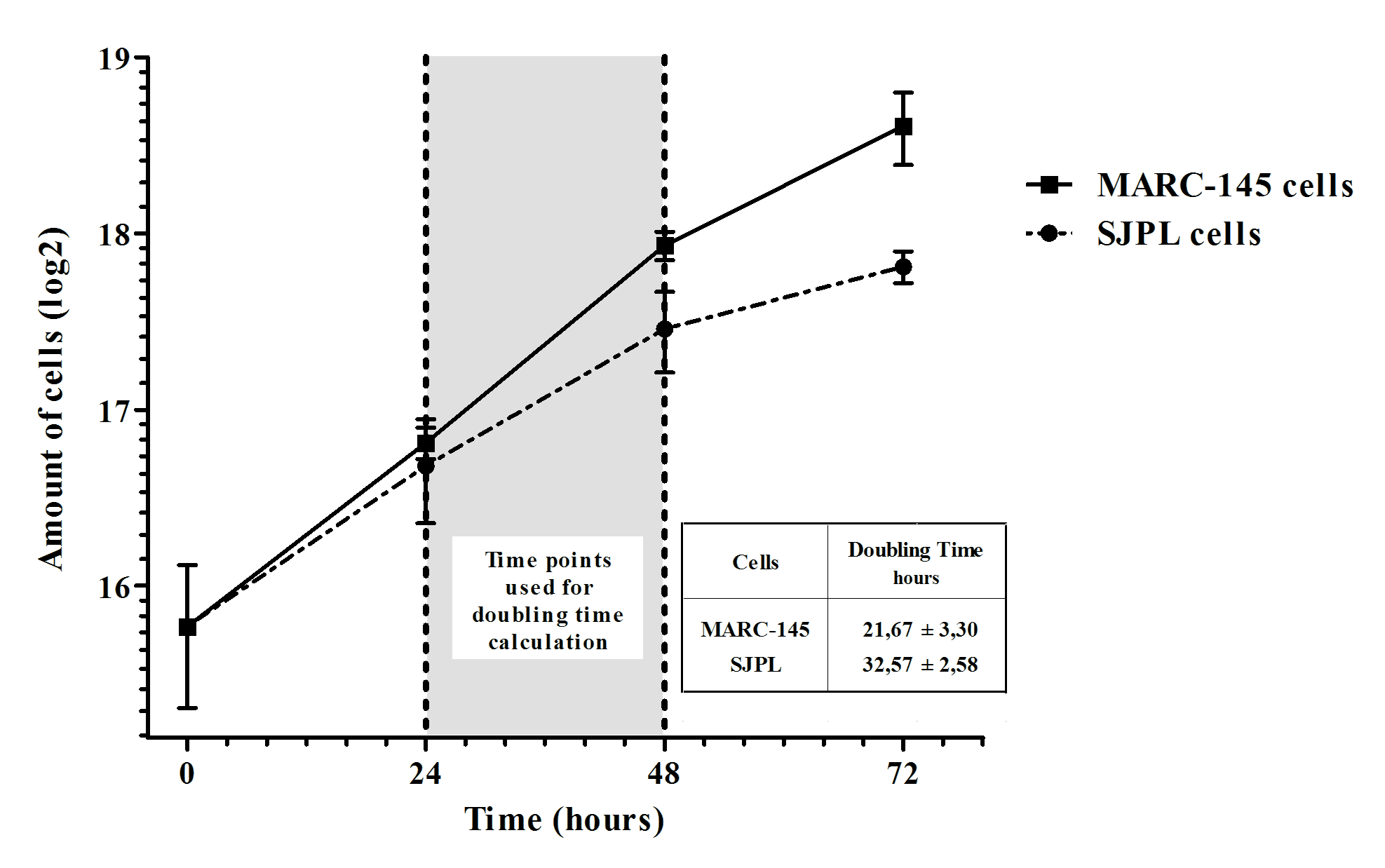

Supplement: Additional file 1 — Figure S1. SJPL and MARC-145 cells growth curves. Amount of cells (log2) was calculated with a hemacytometer at seeding time (0 hr), and at 24, 48, and 72 hrs post-incubation. Doubling time was calculated with data obtained at 24 and 48 hrs post-incubation with the formula: Doubling time (hrs) = 0.3 X incubation time (hrs) / (log cell time b- log cell time a), where incubation time is 24 hrs, cell time a is the amount of cells at 24 hrs and cell time b is the amount of cells at 48 hrs. Interestingly, the MARC-145 cells grow faster than the SJPL cells. During this experiment, cells surface areas were calculated with ImageJ v.1.6.0 from three different pictures of each cell type at 24 hrs post-incubation. SJPL and MARC-145 cells have a surface area of 4684.41 ± 2188.94 μm2 (n = 29) and 3568.96 ± 1128.47 μm2 (n = 38), respectively. Statistical analyses using t test showed that SJPL cells have a statistically wider surface than MARC-145 cells (P < 0.01) (data not shown). [file 1743-422X-9-267-S1.tiff]
